# Effects of different statin-based lipid-lowering therapies on stabilization and regression of carotid plaque: a randomized open-label trial

**DOI:** 10.3389/fphar.2026.1841158

**Published:** 2026-08-27

**Authors:** Sihua Luo, Lili Lin, Yuhui Lin, Pengda Qiu, Kuan Cai, Huanliang Huang, Yidi Zeng, Hanhui Huang, Hao Liang, Yunhong Xu

**Affiliations:** 1 Department of General Medicine, Guangdong Provincial Key Laboratory of Major Obstetric Diseases, Guangdong Provincial Clinical Research Center for Obstetrics and Gynecology, The Third Affiliated Hospital of Guangzhou Medical University, Guangzhou, China; 2 Department of Cardiology, Guangdong Provincial Key Laboratory of Major Obstetric Diseases, Guangdong Provincial Clinical Research Center for Obstetrics and Gynecology, The Third Affiliated Hospital of Guangzhou Medical University, Guangzhou, China; 3 Department of Cardiovascular Medicine, Liwan Central Hospital of Guangzhou, Guangzhou, China; 4 Department of Ultrasound, Guangdong Provincial Key Laboratory of Major Obstetric Diseases, Guangdong Provincial Clinical Research Center for Obstetrics and Gynecology, The Third Affiliated Hospital of Guangzhou Medical University, Guangzhou, China; 5 Institute of TCM Diagnostics, Hunan University of Chinese Medicine, Changsha, China; 6 Department of Pharmacy, Guangdong Provincial Key Laboratory of Major Obstetric Diseases, Guangdong Provincial Clinical Research Center for Obstetrics and Gynecology, The Third Affiliated Hospital of Guangzhou Medical University, Guangzhou, China

**Keywords:** atherosclerotic plaque, contrast-enhanced carotid ultrasonography, intraplaque neovascularization, PCSK9 inhibitor, plaque regression

## Abstract

**Background:**

Atherosclerotic plaques in the carotid arteries are significant risk factors for cardiovascular events. This study aimed to evaluate the efficacy of different statin-based lipid-lowering therapies, including statins and the addition of ezetimibe or PCSK9 inhibitors, in stabilizing and regressing carotid plaques in a high cardiovascular risk population.

**Methods:**

In this randomized open-label trial, participants were randomly assigned to one of four treatment groups: atorvastatin 20 mg/day (Statin), atorvastatin 10 mg/day + ezetimibe 10 mg/day (Statin_E), atorvastatin 10 mg/day + alirocumab 75 mg every 2 weeks (Statin_P), and atorvastatin 10 mg/day + ezetimibe 10 mg/day + alirocumab 75 mg every 2 weeks (Statin_EP). The primary outcomes were carotid plaque regression or progression at 12 months. Secondary outcomes included plaque morphological stabilization assessed by contrast-enhanced ultrasound (CEUS) and changes in blood lipid levels after 6 months.

**Results:**

At 6 months, the Statin_P and Statin_EP groups exhibited significantly lower LDL-C levels compared to the Statin and Statin_E groups (p < 0.05). The proportion of intraplaque neovascularization (IPN) scales were significantly different (p = 0.02, CI 95% [0.00, 0.12]) between the groups after treatment, and the IPN was prominently reduced in the Statin_P and Statin_EP groups. At 12 months, Statin_P and Statin_EP groups showed the greatest reduction in carotid plaque size, with significant differences in plaque regression across all treatment groups (p < 0.05, p = 2.65e−08, CI 95% [0.04, 1.00]). The highest proportions of plaque regression were observed in the Statin_P and Statin_EP groups (p < 0.05, p = 1.76e−07, CI 95% [0.09, 0.22]).

**Conclusion:**

The combination of moderate-dose statins with PCSK9 inhibitors is significantly more effective than statin monotherapy or statin-ezetimibe combinations in stabilizing and regressing atherosclerotic carotid plaques. These findings suggest that statin-based combination lipid-lowering therapies offer substantial potential for reducing cardiovascular risk in high-risk Asian populations.

**Trial Registration:**

The trial was registered in the Chinese Clinical Trial Registry (No. ChiCTR2200058389) on 2022-04-08.

## 1 Introduction

Even in asymptomatic individuals, the extent of atherosclerotic burden in the carotid arteries is predictive of adverse cardiovascular events, including stroke and myocardial infarction (Basili et al., 2017). The formation of these plaques is a complex process involving lipid accumulation, inflammatory responses, and endothelial dysfunction within the arterial walls (Toscano et al., 2022). Plaque regression and stabilization are important goals in the management of carotid artery disease, as they can reduce the risk of complications such as thromboembolism and rupture. Atherosclerotic plaques, particularly within the carotid arteries, are well-established markers of cardiovascular risk in the general population.

Lipid-lowering therapies, particularly statins, have been the cornerstone of preventing atherosclerotic cardiovascular disease for decades. Recently, their role in inducing plaque regression and promoting plaque stabilization has emerged as a pivotal objective in managing cardiovascular risk. The ASTEROID trial (Chhatriwalla et al., 2006) demonstrated that high-dose rosuvastatin could promote the regression of coronary atheroma assessed by intravascular ultrasound (IVUS). Normally, only by intensive lipid-lowering therapy (either through high-dose statins or combination therapy) to reduce lipid to a target low level can the effect of plaque regression be achieved, but studies have shown that Asians, particularly Chinese patients, achieve greater LDL-C reductions with lower doses of statins compared to Western populations, due to differences in pharmacokinetics (Dai et al., 2016). Moderate-intensity statin therapy, such as atorvastatin 20 mg, is called the “Chinese dose” statins because of compromise to balance effectiveness and safety in the Chinese population (Ye, 2016). The Chinese guidelines for dyslipidemia management consistently support the use of moderate-intensity statin therapy (Li et al., 2023). When moderate-intensity statin cannot satisfy the intensive lipid-lowering requirements for high-risk patients, statin combination with other agents is recommended. The PRECISE-IVUS trial (Tsujita et al., 2015) showed that a combination of statins and ezetimibe achieved greater coronary plaque regression compared to standard statin monotherapy. Seminal atorvastatin studies predate this work: the REVERSAL trial (Schoenhagen et al., 2006) demonstrated that intensive therapy with atorvastatin 80 mg/day halted the progression of coronary atherosclerosis measured by IVUS, compared with pravastatin 40 mg/day, and the PROVE-IT trial (Cannon et al., 2004) showed that high-dose atorvastatin reduced major cardiovascular events after acute coronary syndrome compared with standard-dose pravastatin, establishing intensive atorvastatin therapy as the cornerstone for plaque stabilization and event reduction.

As a novel class of lipid-lowering agents, proprotein convertase subtilisin/kexin type 9 (PCSK9) inhibitors can significantly reduce low-density lipoprotein cholesterol (LDL-C) levels. The addition of the PCSK9 inhibitor evolocumab to statin therapy has also been shown to reverse atherosclerotic plaque (Nicho et al., 2016). In addition, studies have shown that PCSK9 inhibitors can reduce intraplaque neovascularization (IPN) within plaques (Lepor et al., 2021), which is an important factor in plaque progression and vulnerability. Ultrasound imaging techniques, such as contrast-enhanced ultrasonography (CEUS), can evaluate intraplaque vessels and the vasa vasorum. Several studies have verified the presence of IPN using CEUS, indicating that it is a suitable modality for detecting plaque stability (Yan et al., 2021).

Our previous study showed that intensive therapy with a statin and a PCSK9 inhibitor was more effective for lowering lipid levels of patients at high cardiovascular disease (CVD) risk compared to statin alone or statin plus ezetimibe (Lin et al., 2023). However, the effect on regression and stabilization of carotid plaques remains unknown. CEUS has enabled the accurate quantification and characterization of atherosclerotic plaques, providing an opportunity to directly monitor the impact of therapeutic interventions on plaques. Therefore, we present a nonblind, randomized controlled trial to compare the effects of different “Chinese Dose” lipid-lowering therapies on the regression and stabilization of carotid plaques in patients at high CVD risk.

## 2 Methods

### 2.1 Study population

Patients were enrolled from the Third Affiliated Hospital of Guangzhou Medical University from March 2022 to December 2023. This study was part of the trial registered in the Chinese Clinical Trial Registry (registration number: ChiCTR2200058389). The study was conducted and reported in accordance with the CONSORT 2010 guidelines. The study protocol was approved by the Institutional Ethics Committee of the Third Affiliated Hospital of Guangzhou Medical University (approval number: 2022-027), and written informed consent was obtained from all patients before the treatment.

Study participants with high CVD risk (Mach et al., 2019) with non-calcified plaque on the carotid artery were included based on the following criteria: (1) treatment-naive or receiving low-dose statin therapy (e.g., atorvastatin 10 mg, simvastatin 20 mg), or ezetimibe monotherapy that result in an LDL-C reduction of less than 50%; (2) LDL-C ≥70 mg/dL and not previously receiving high-intensity statin therapy or combination therapy with PCSK9 inhibitors or ezetimibe; (3) uni- or bilateral carotid artery plaque (maximum plaque thickness >1.5 mm). Individuals with a history of stroke and myocardial infarction; those with a history of carotid endarterectomy; those with an allergy to the contrast agent or lipid-lowering drugs; those with secondary dyslipidemia caused by hypothyroidism, Cushing’s syndrome, nephrotic syndrome, or drug use; and those with heart failure or end-stage renal failure were excluded from the study.

### 2.2 Study design

Information on patients’ sex, age, body mass index (BMI), smoking history, hypertension, diabetes, and other related medical history was collected. Baseline laboratory tests and carotid ultrasonography were performed, and patients who met the inclusion criteria were enrolled. The randomization and allocation procedures were carried out as before (Lin et al., 2023). In short, an assistant used random numbers obtained from sealed envelopes. All selected patients were assigned (1:1:1:1) to one of the following groups: 1) atorvastatin 20 mg/d or its equivalent (Statin) group, 2) atorvastatin 10 mg/d or its equivalent + ezetimibe 10 mg/d (Statin_E) group, 3) atorvastatin 10 mg/d or its equivalent + alirocumab 75 mg/2 weeks (Statin_P) group, and 4) atorvastatin 10 mg/d or its equivalent + ezetimibe 10 mg/d + alirocumab 75 mg/2 weeks (Statin_EP) group. Follow-up ultrasonography was performed every 6 months after baseline, and lipid panel, including total cholesterol (TC), LDL-C, triglycerides (TG), and high-density lipoprotein cholesterol (HDL-C), was measured every 3 months. All eligible patients received continuous standard treatments for underlying conditions, along with recommendations for dietary modifications and lifestyle management. These recommendations encompassed abstaining from smoking and alcohol consumption, adhering to a low-fat diet, and participating in appropriate physical exercise. The patients were followed up for 12 months.

### 2.3 Carotid ultrasonography

The patient was requested to lie in a supine position with their head slightly extended. The neck was slightly rotated to the opposite side of the carotid artery being examined to allow better visualization of the artery. Standard carotid ultrasound and CEUS examinations were performed for all patients using a Mindray Resona 8T Ultrasound Machine (Mindray Bio-Medical Electronics Co., Ltd., Shenzhen, China) with a 9–11-MHz linear transducer (Mindray L9-3U). The artery and plaque were examined in longitudinal and transverse planes utilizing standard ultrasound techniques. The degree of carotid artery stenosis was assessed based on peak-systolic and end-diastolic velocities, following the consensus criteria established by the Society of Radiologists in Ultrasound (Grant et al., 2003). All plaques were scanned dynamically in grayscale mode to measure maximum plaque thickness and length, location, echogenicity, and mobility. The target plaque for serial follow-up was predefined as the thickest plaque identified at the baseline scan on each side (left/right common, bifurcation, internal and external carotid segments). At each follow-up visit, the same plaque was re-identified using anatomical landmarks (carotid bifurcation, calcified reference points and vessel curvature) and matched-plane greyscale stills were recorded. Plaques with dense posterior acoustic shadowing from heavy calcification that precluded reliable border delineation on either the baseline or follow-up scan were excluded from the size analysis, and any new plaque that did not meet the >1.5 mm thickness criterion was not used as the index lesion.

The CEUS was carried out by injecting a 2-mL bolus of microbubble agent SonoVue (Bracco Imaging, Milan, Italy) via the median cubital vein, followed by a 5-mL normal saline flush. The contrast-specific imaging mode was set at a low mechanical index (−0.12) to prevent the destruction of contrast microbubbles (measuring 1–11 μm). Cine clips were recorded in the longitudinal plane for 10–20 s at baseline and continued for up to 100 s after the microbubbles reached the arteries. The raw clips were stored for further assessment offline. All procedures were performed by a vascular sonographer with more than 10 years of experience, who was blinded to the clinical characteristics of the participants. CEUS was performed on each participant at least twice (baseline and 6 months) during the trial.

### 2.4 Image analysis

Intima-media thickness (IMT) was measured on the common carotid artery, and maximum plaque height was defined as the maximum distance from the intima-lumen interface to the media-adventitia interface. Atherosclerotic plaques were defined as focal structures encroaching into the arterial lumen with a height >1.5 mm. The degree of carotid artery stenosis was determined based on peak-systolic and end-diastolic velocities according to the consensus criteria of the Society of Radiologists in Ultrasound (Grant et al., 2003). The maximal plaque thickness, calcified area, and hypoechoic area were measured on the thickest cross-section of the plaque. Morphological features of the carotid plaques were categorized according to plaque echogenicity as follows: type I: predominantly hypoechoic with a thin echogenic rim; type II: echogenic plaque with >50% hypoechoic areas; type III: echogenic plaque with <50% hypoechoic areas; and type IV: uniformly echogenic plaque. Plaque size was defined as length (mm) × maximum thickness (mm), measured on longitudinal and transverse sections respectively. For patients with multiple plaques, the largest plaque on each side was identified and measured (yielding a maximum of 2 plaques per patient). The target plaque for serial follow-up was predefined as the thickest plaque identified at the baseline scan on each side (left/right common, bifurcation, internal and external carotid segments). At each follow-up visit, the same plaque was re-identified using anatomical landmarks (carotid bifurcation, calcified reference points and vessel curvature) and matched-plane greyscale stills were recorded. Plaques with dense posterior acoustic shadowing from heavy calcification that precluded reliable border delineation on either the baseline or follow-up scan were excluded from the size analysis, and any new plaque that did not meet the >1.5 mm thickness criterion was not used as the index lesion.

Intraplaque contrast enhancement was semi-quantitatively graded using the following scales: IPN = 0, no visible microbubbles within the plaque; IPN = 1, mild microbubbles confined to the shoulder and/or adventitial side of the plaque; IPN = 2, linear microbubbles extending into the plaque; and IPN = 3, extensive microbubbles throughout the plaque (Shah et al., 2007).

The data were analyzed by two experienced sonographers in CEUS, who were blinded to the clinical information of the patients and each other’s results. Any inconsistent results were discussed, and the final report was determined by consensus. To assess the reliability of CEUS interpretations, we calculated the intraclass correlation coefficient (ICC) between the two sonographers for both IPN grading and plaque echogenicity assessment. The ICC for inter-observer agreement was 0.79, and intra-observer variability was assessed by re-evaluating a subset of images, yielding an ICC of 0.91. Readers were additionally blinded to treatment allocation, chronological order (baseline vs. follow-up) and patient identity; all images were anonymized, de-identified and re-ordered by a study coordinator using a computer-generated random sequence prior to reading.

### 2.5 Outcome measures

The primary measures of the study were: (1) The secondary measures were: (1) carotid plaque morphological stabilization based on plaque echogenicity and IPN categories from CEUS at 6-month follow-up, and (2) changes in blood lipid levels after 6 months. Change of maximum carotid plaque thickness (mm) measured on longitudinal B-mode images at the thickest cross-section of each plaque, and (2) regression or progression at 12 months, defined as a reduction or increment in maximum plaque thickness of at least 10% relative to baseline. The 10% threshold exceeds the mean intra-observer coefficient of variation (CV) for repeated maximum-thickness measurements obtained from a pilot set of 30 plaques (CV = 4.8%) and the inter-observer CV (6.2%), as reflected in the reported inter- and intra-observer ICCs of 0.79 and 0.91, respectively, so a 10% change is unlikely to reflect measurement noise.

### 2.6 Sample size calculation

The sample size for this study was calculated using Chi-Square Tests to compare four treatment groups. Considering a drop out of 15% based on data from our preliminary test, the occurrence of the primary endpoint (plaque regression) in 20%, 21%, 35%, 34% of patients in Statin, Statin_E, Statin_P, and Statin_EP groups, an effect size of 0.157 was assumed. To achieve 80% power with a two-sided alpha of 0.05, a minimum of 520 carotid plaques was required. To account for potential dropouts and loss to follow-up, we enrolled 312 patients, of whom 299 with 538 carotid plaques completed the study. The sample size calculation was performed using the Power Analysis and Sample Size Software (PASS, 2023 v23.0.2, Utah, USA). A limitation of this calculation is that it was performed at the plaque level rather than the patient level, despite randomisation occurring at the patient level; without an *a-priori* assumed intraclass correlation coefficient (ICC) for plaque responses within patients, the corresponding effective patient-level sample size is smaller than the 299 patients retained, and the patient-level power of the present trial is therefore limited. This is explicitly acknowledged in the Limitations section.

### 2.7 Statistical analysis

R software (ver. 4.2.1, The R Foundation, Vienna, Austria) was used for statistical analysis. Normally distributed data are presented as the mean ± standard deviation (SD). One-way ANOVA was performed for comparisons of multiple groups, and t-tests were used for comparisons between two groups. Non-normally distributed data are presented as the median [25th percentile, 75th percentile], and Kruskal–Wallis one-way ANOVA was performed for comparisons between multiple groups. A nonparametric test (Dunn’s test) with P value adjustment (Holm method) was used for pairwise comparisons. Categorical data are presented as frequencies or percentages, and Chi-square test or Kruskal–Wallis test (ordered categorical data) were applied for comparisons between groups. McNemar-Bowker test was used to assess within-group categorical data regarding changes of plaque vulnerability (baseline and post-treatment grading). A p-value of <0.05 was considered statistically significant. For between-group treatment comparisons, the primary inferential framework was the comparison of 6- and 12-month changes across the four randomised arms, with adjustment for the corresponding baseline value (analysis of covariance for continuous endpoints; ordinal regression/proportional-odds shift models for the ordinal IPN and echogenicity outcomes). Because 299 patients contributed 538 plaques and 80.9% had bilateral disease, observations from the same patient are not independent; a sensitivity analysis using mixed-effects models (random patient intercept; plaque nested within patient) and generalised estimating equations (GEE) with an exchangeable working correlation structure was therefore prespecified to re-estimate the treatment effect on plaque thickness, echogenicity and IPN, accounting for within-patient clustering. The McNemar-Bowker test was retained only as a descriptive within-group summary.

## 3 Results

### 3.1 Study flow and basic characteristics

**FIGURE 1:**
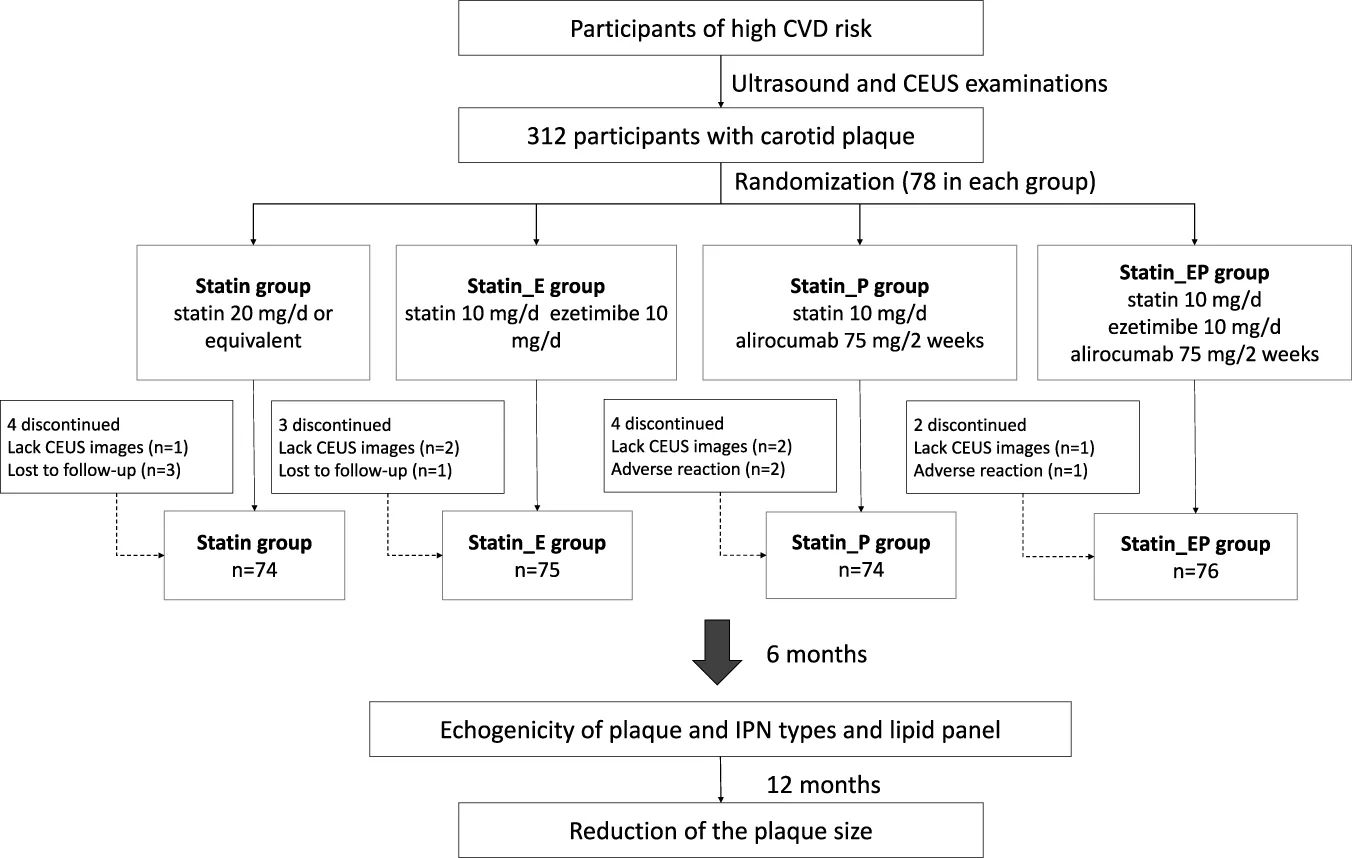
Flowchart of the study.

In total, 312 patients (78 in each group) who met the criteria were initially enrolled. Of these, 13 patients were excluded because of insufficient CEUS images, loss to follow-up, and adverse reactions. Ultimately, 299 patients (74 in the Statin group, 75 in the Statin_E group, 74 in the Statin_P group, and 76 in the Statin_EP group) were included in this study (Figure 1). The basic characteristics of the participants in the four groups are summarized in Table 1. No statistically significant differences were found in terms of sex, age, BMI, hypertension, blood glucose, and lipid levels between the groups. A CONSORT-style flow diagram with the number of patients screened, enrolled, randomly assigned, excluded after randomisation (with reasons stratified by treatment arm, distinguishing imaging failure from treatment discontinuation or adverse reactions) and analysed is provided in Figure 1.

**TABLE 1:** Basic characteristics of the participants.

Variable	N	Statin, N = 74^1^	Statin_E, N = 75^1^	Statin_P, N = 74^1^	Statin_EP, N = 76^1^	p-value^2^
Sex	299	​	​	​	​	0.41
Female	​	42/57%	40/53%	38/51%	33/43%	​
Male	​	32/43%	35/47%	36/49%	43/57%	​
Age (y)	299	73 ± 9	71 ± 9	71 ± 10	70 ± 10	0.28
BMI (kg/m^2^)	299	23.3 ± 3.1	23.7 ± 3.6	23.8 ± 3.5	23.9 ± 2.9	0.77
HT (mmHg)	299	66/89%	66/88%	61/82%	58/76%	0.12
Smoking	299	19/26%	21/28%	22/30%	29/38%	0.37
T2DM	299	33/45%	31/41%	36/49%	33/43%	0.84
FPG (mmol/L)	299	6.3 [5.4, 9.1]	6.1 [5.2, 8.5]	6.5 [5.5, 9.3]	6.3 [5.3, 8.6]	0.82
HbA1c (%)	299	6.25 [5.90, 6.80]	6.20 [5.90, 6.75]	6.40 [5.80, 7.20]	6.25 [5.90, 6.80]	0.95
TC (mmol/L)	299	3.86 [3.46, 4.57]	4.13 [3.43, 5.08]	3.87 [3.56, 4.50]	4.22 [3.25, 5.03]	0.31
TG (mmol/L)	299	1.37 [0.98, 1.80]	1.49 [0.96, 2.08]	1.46 [1.05, 2.15]	1.27 [0.99, 1.71]	0.41
LDL-C (mmol/L)	299	2.35 [1.87, 2.95]	2.67 [1.96, 3.61]	2.37 [2.02, 2.79]	2.56 [1.88, 3.46]	0.23
HDL-C (mmol/L)	299	1.16 [0.90, 1.52]	1.11 [0.91, 1.46]	1.12 [0.93, 1.32]	1.21 [1.04, 1.48]	0.11
Scr (μmol/L)	299	91 [72, 115]	81 [68, 99]	82 [70, 104]	81 [68, 101]	0.15
UA (μmol/L)	299	359 [307, 474]	381 [321, 458]	393 [335, 467]	384 [315, 431]	0.35

1 n/%; Mean ± SD; Median (IQR).

2 Pearson’s Chi-squared test; One-way ANOVA; Kruskal–Wallis rank sum test.

BMI, body mass index; FPG, fasting plasma glucose, HbA1c = Glycated Hemoglobin, HDL-C, High-Density Lipoprotein Cholesterol; LDL-C, Low-Density Lipoprotein Cholesterol, Scr = Serum Creatinine, TC, total cholesterol; TG, triglycerides; UA = uric acid.

### 3.2 Lipid profiles after treatment

**FIGURE 2:**
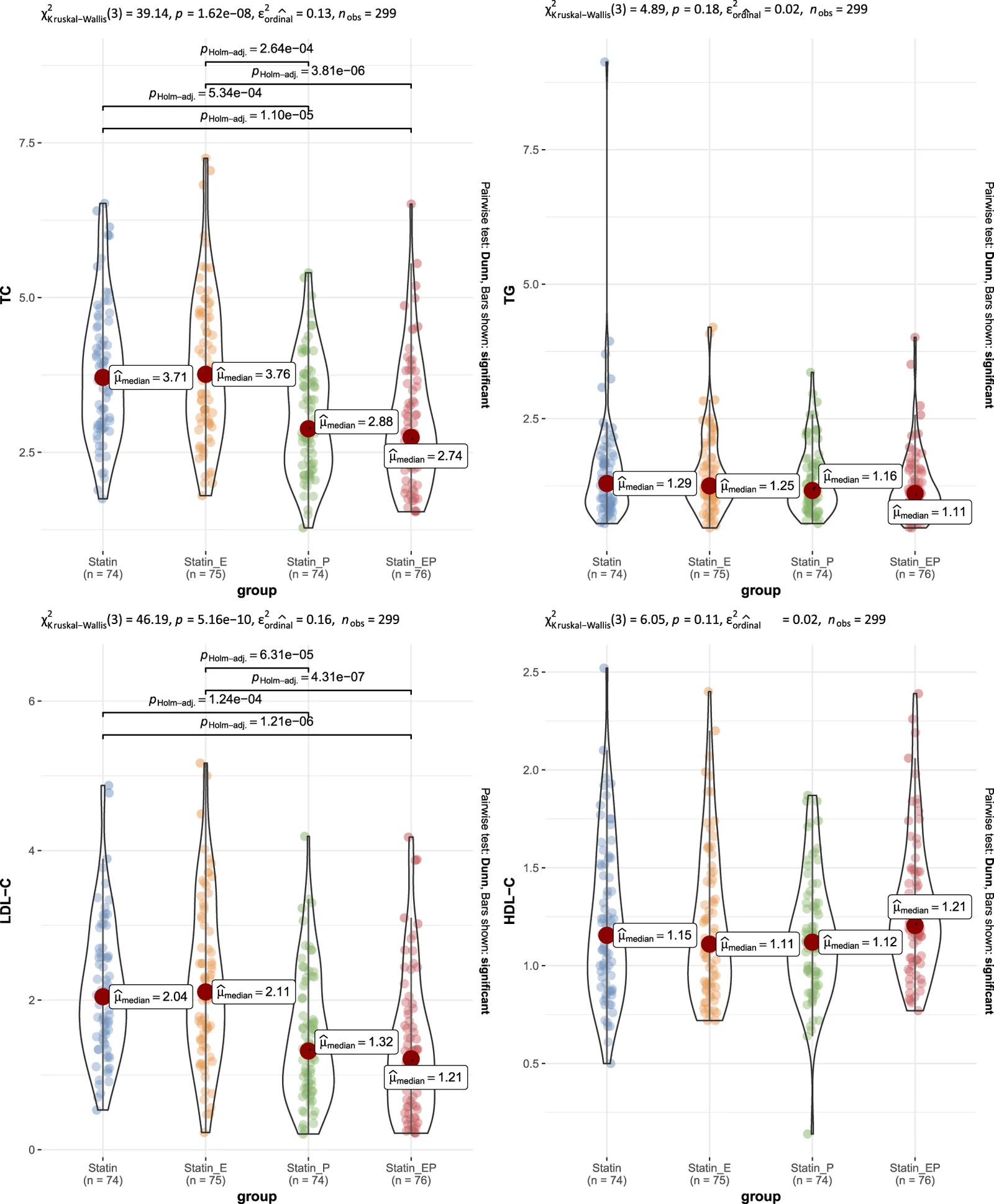
Lipid profiles of the groups after 6 months of treatment.

After 6 months of treatment, TC and LDL-C levels significantly differed among the four groups (Figure 2). Pairwise comparisons showed that the levels of TC and LDL-C in the Statin_P group and the Statin_EP group were significantly lower than those in the Statin group and Statin_E group. However, there were no significant differences in TC and LDL-C levels between the Statin_P group and the Statin_EP group. HDL-C and TG levels were more favorable in the Statin_EP group, but the differences were not statistically significant at 6 months.

### 3.3 Baseline plaque characteristics

Of the 299 patients, plaques of bilateral carotid arteries were found in 239 (80.0%) patients, while plaques of unilateral carotid artery were found in 60 (20.0%) patients. A total of before treatment. There were no significant differences in plaque sizes and IMT between the four groups before treatment. Moreover, the Kruskal–Wallis test did not show any significant differences in the proportions of plaque echogenicity types and IPN scales (Table 2). 538 plaques were recorded.

**TABLE 2:** Baseline plaque characteristics of ultrasound imaging.

Variable	N	Statin, N = 74^1^	Statin_E, N = 75^1^	Statin_P, N = 74^1^	Statin_EP, N = 76^1^	p-value^2^
LIMT (mm)	299	0.92 ± 0.15	0.97 ± 0.17	0.96 ± 0.20	0.97 ± 0.21	0.28
RIMT (mm)	299	0.91 ± 0.16	0.95 ± 0.18	0.93 ± 0.18	0.95 ± 0.20	0.44
L-type	275	​	​	​	​	0.67
I	​	6/72 [8.3%]	11/70 [16%]	10/68 [15%]	6/65 [9.2%]	​
II	​	30/72 [42%]	25/70 [36%]	29/68 [43%]	26/65 [40%]	​
III	​	29/72 [40%]	29/70 [41%]	23/68 [34%]	27/65 [42%]	​
IV	​	7/72 [9.7%]	5/70 [7.1%]	6/68 [8.8%]	6/65 [9.2%]	​
R-type	263	​	​	​	​	0.62
I	​	9/64 [14%]	7/68 [10%]	8/65 [12%]	8/66 [12%]	​
II	​	21/64 [33%]	20/68 [29%]	22/65 [34%]	25/66 [38%]	​
III	​	28/64 [44%]	31/68 [46%]	29/65 [45%]	26/66 [39%]	​
IV	​	6/64 [9.4%]	10/68 [15%]	6/65 [9.2%]	7/66 [11%]	​
*Bilateral type*	538	​	​	​	​	0.83
I	​	15/136 [11%]	18/138 [13%]	18/133 [14%]	14/131 [11%]	​
II	​	51/136 [38%]	45/138 [33%]	51/133 [38%]	51/131 [39%]	​
III	​	57/136 [42%]	60/138 [43%]	52/133 [39%]	53/131 [40%]	​
IV	​	13/136 [9.6%]	15/138 [11%]	12/133 [9.0%]	13/131 [9.9%]	​
L-neo	275	​	​	​	​	0.87
L0	​	33/72 [46%]	29/70 [41%]	34/68 [50%]	33/65 [51%]	​
L1	​	19/72 [26%]	22/70 [31%]	16/68 [24%]	15/65 [23%]	​
L2	​	14/72 [19%]	13/70 [19%]	11/68 [16%]	11/65 [17%]	​
L3	​	6/72 [8.3%]	6/70 [8.6%]	7/68 [10%]	6/65 [9.2%]	​
R-neo	263	​	​	​	​	0.78
R0	​	32/64 [50%]	35/68 [51%]	31/65 [48%]	36/66 [55%]	​
R1	​	14/64 [22%]	13/68 [19%]	10/65 [15%]	12/66 [18%]	​
R2	​	12/64 [19%]	12/68 [18%]	14/65 [22%]	10/66 [15%]	​
R3	​	6/64 [9.4%]	8/68 [12%]	10/65 [15%]	8/66 [12%]	​
*Bilateral neo*	538	​	​	​	​	0.85
0	​	65/136 [48%]	64/138 [46%]	65/133 [49%]	69/131 [53%]	​
1	​	33/136 [24%]	35/138 [25%]	26/133 [20%]	27/131 [21%]	​
2	​	26/136 [19%]	25/138 [18%]	25/133 [19%]	21/131 [16%]	​
3	​	12/136 [8.8%]	14/138 [10%]	17/133 [13%]	14/131 [11%]	​

1 Mean ± SD; median (IQR); n/N (%).

2 One-way ANOVA; Kruskal–Wallis test.

Bilateral neo, bilateral intraplaque neovascularization; bilateral type, bilateral plaque echogenic type, LIMT, left intima-media thickness, L-neo, left intraplaque neovascularization, L-size, left plaque size, L-type, left plaque echogenic type, RIMT, right intima-media thickness, R-neo, right intraplaque neovascularization, R-size, right plaque size, R-type, right plaque echogenic type.

### 3.4 Changes in carotid plaque characteristics

**FIGURE 3:**
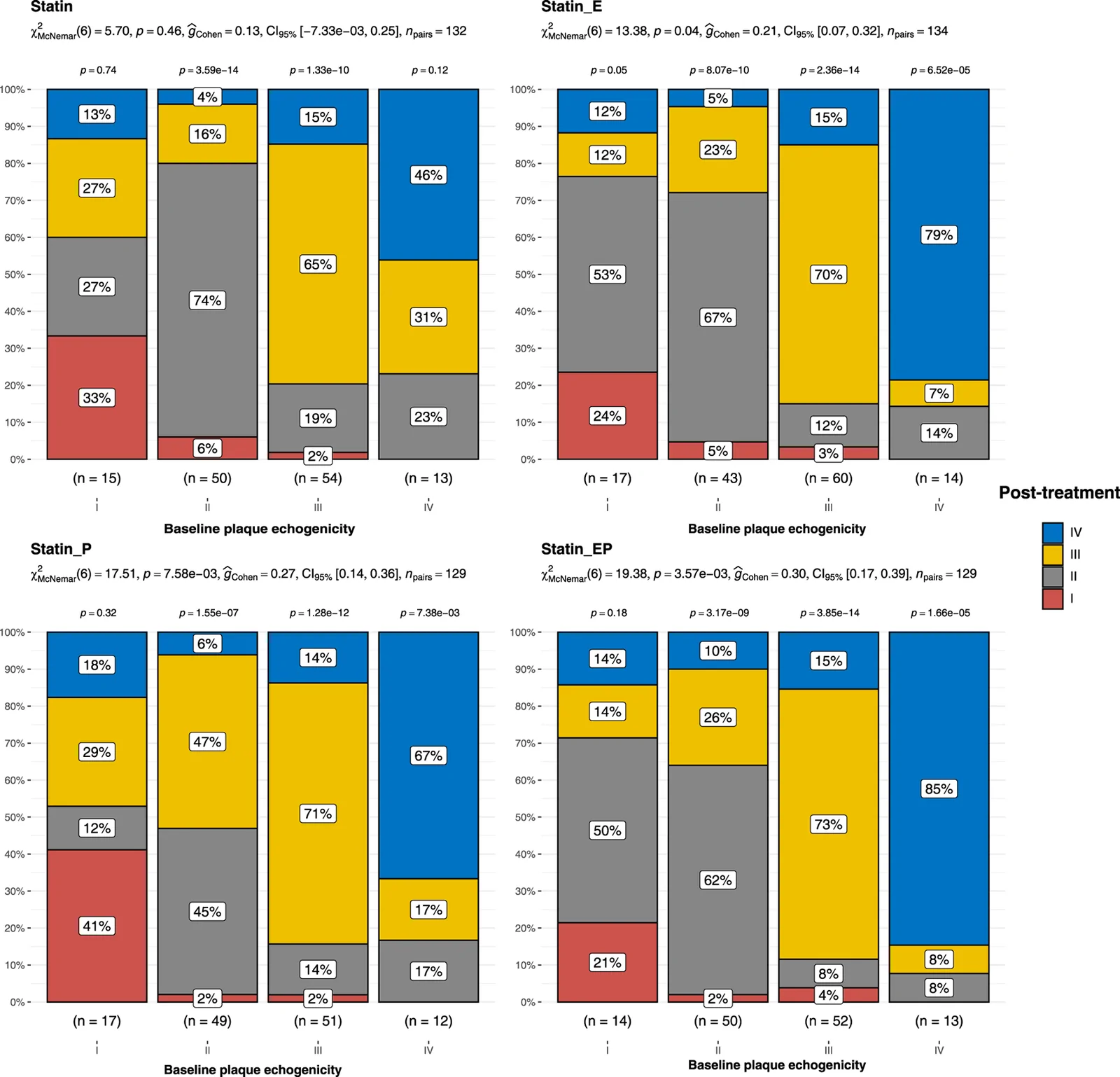
Within-group comparisons of pre- and post-treatment bilateral plaque echogenicity types.

**FIGURE 4:**
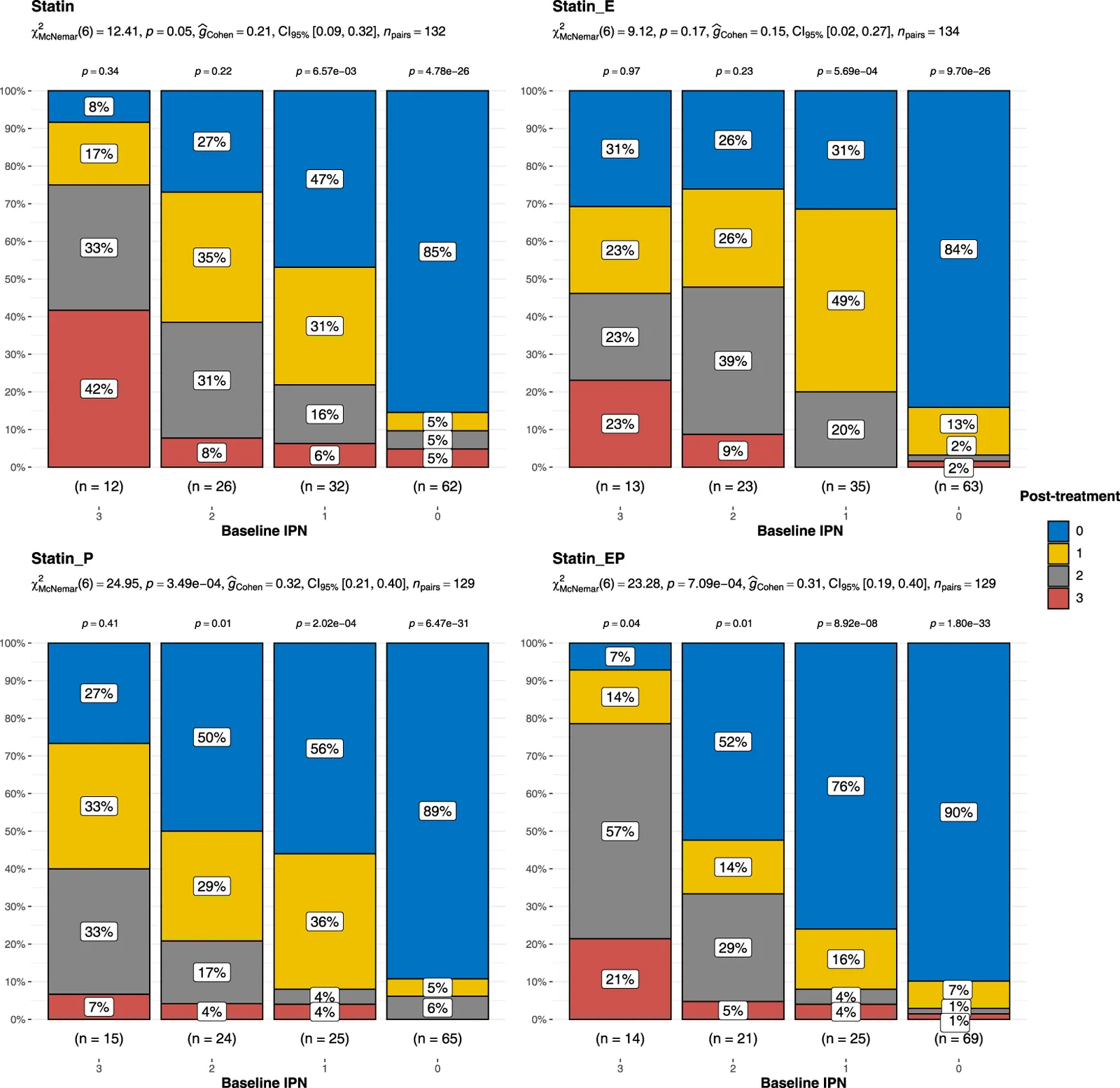
Within-group comparisons of pre- and post-treatment bilateral intraplaque neovascularization scales.

Within-group comparisons (Baseline vs. post-treatment) of bilateral plaque echogenicity showed that the types of plaque echogenicity were significantly different in the Statin_E (p = 0.04, gCohen = 0.21, CI 95% [0.07, 0.32], Figure 3), Statin_P (p = 7.58e−03, gCohen = 0.27, CI 95% [0.14, 0.36], Figure 3) and Statin_EP (p = 3.57e−03, gCohen = 0.30, CI 95% [0.17, 0.39], Figure 3) groups. The proportion of bilateral IPN scales significantly differed before and after treatment in the Statin_P (p = 3.49e−04, 0.32, CI 95% [0.21, 0.40], Figure 4) and Statin_EP group (p = 0.008, gCohen = 0.413, CI 95% [0.232, 0.476], Figure 4).

**FIGURE 5:**
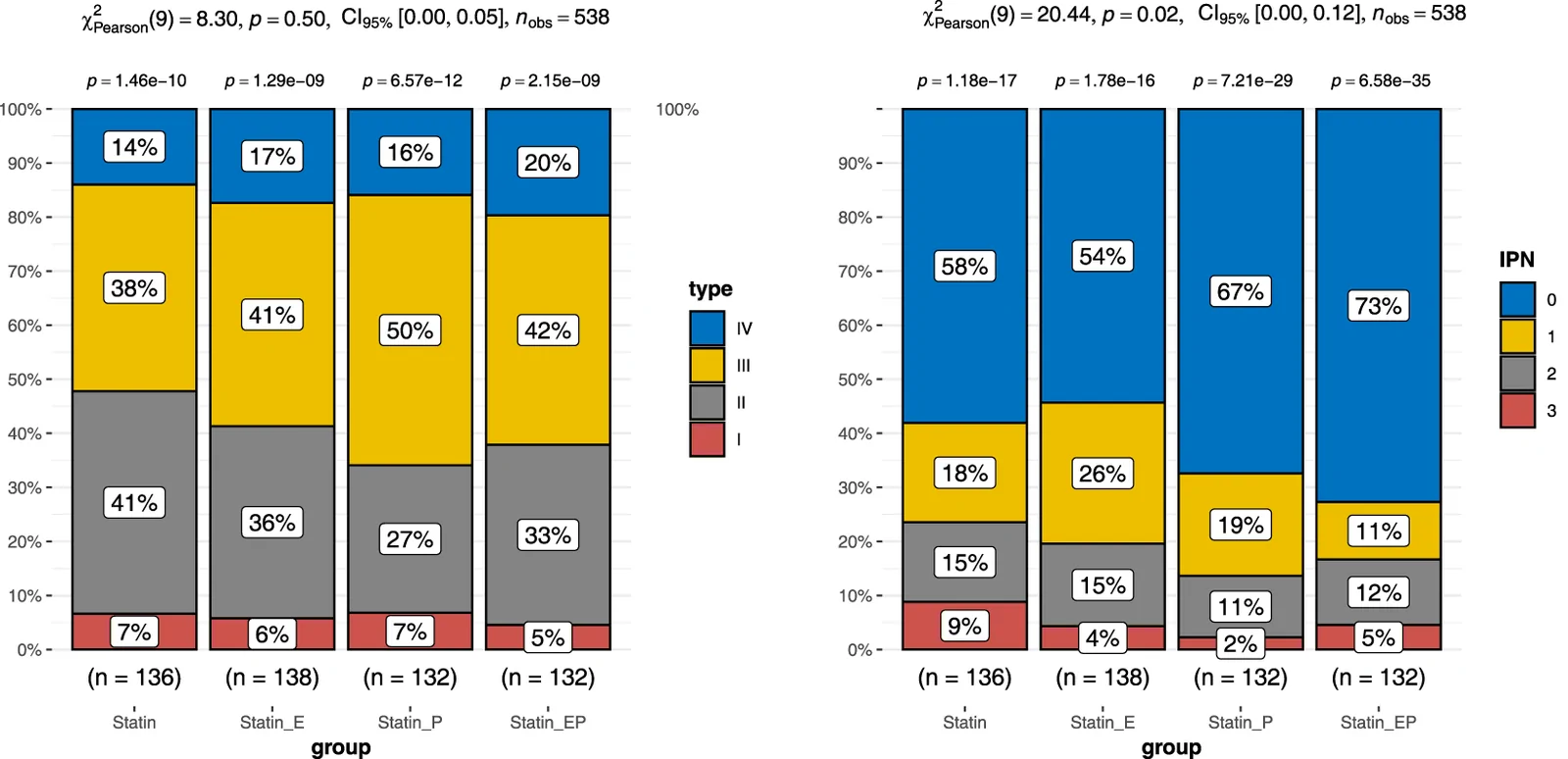
Proportions of bilateral plaque echogenicity types and intraplaque neovascularization scales in the four groups after treatment.

The proportions of 0 grade IPN were higher in the Statin_P and Statin_EP groups after treatment. However, the proportions of plaque echogenicity types after treatment showed no significant difference between the groups. The proportions of bilateral IPN scales were significantly different (p = 0.02, CI 95% [0.00, 0.12], Figure 5) between the groups after treatment.

### 3.5 Changes in carotid plaque sizes

Although no statistically significant differences in plaque sizes were found between the groups before or after treatment, the change of plaque size (post-treatment minus baseline) significantly differed among the four groups (Table 3).

**TABLE 3:** Changes in baseline and post-treatment carotid plaque sizes.

Variable	N	Statin, N = 74^1^	Statin_E, N = 75^1^	Statin_P, N = 74^1^	Statin_EP, N = 76^1^	p-value^2^
*Literal*	​	mm^2^	​	​	​	​
L-size	299	14 [5, 25]	13 [7, 30]	19 [11, 33]	14 [7, 28]	0.11
L-size-post	299	19 [9, 35]	18 [11, 36]	17 [10, 34]	16 [8, 27]	0.20
L-change	299	2 [0, 8]	3 [0, 10]	0 (−4, 3)	0 (−3, 3)	<0.001
R-size	299	14 [7, 27]	14 [8, 25]	17 [9, 29]	17 [10, 32]	0.54
R-size-post	299	17 [10, 33]	17 [9, 27]	17 [6, 32]	17 [9, 24]	0.94
R-change	299	2 [0, 8]	0 [-3, 8]	0 [-3, 2]	0 [-4, 0]	<0.001
*Bilateral*	​	mm^2^	​	​	​	​
Size	598	14 [5, 25]	13 [8, 28]	18 [10, 30]	16 [8, 30]	0.60
Size-post	598	18 [10, 34]	17 [10, 34]	17 [9, 32]	16 [8, 25]	0.15
Change	598	2 [0, 8]	2 [-1, 8]	0 [-4, 2]	0 [-4, 2]	<0.001

1 Median (IQR).

2 Kruskal–Wallis rank sum test.

L-change, left change of baseline and post-treatment plaque size, L-size, left plaque size, L-size-post, left post-treatment plaque size, R-change, right change of baseline and post-treatment plaque size, R-size, right plaque size, R-size-post, right post-treatment plaque size. No plaque: Size = 0.

**FIGURE 6:**
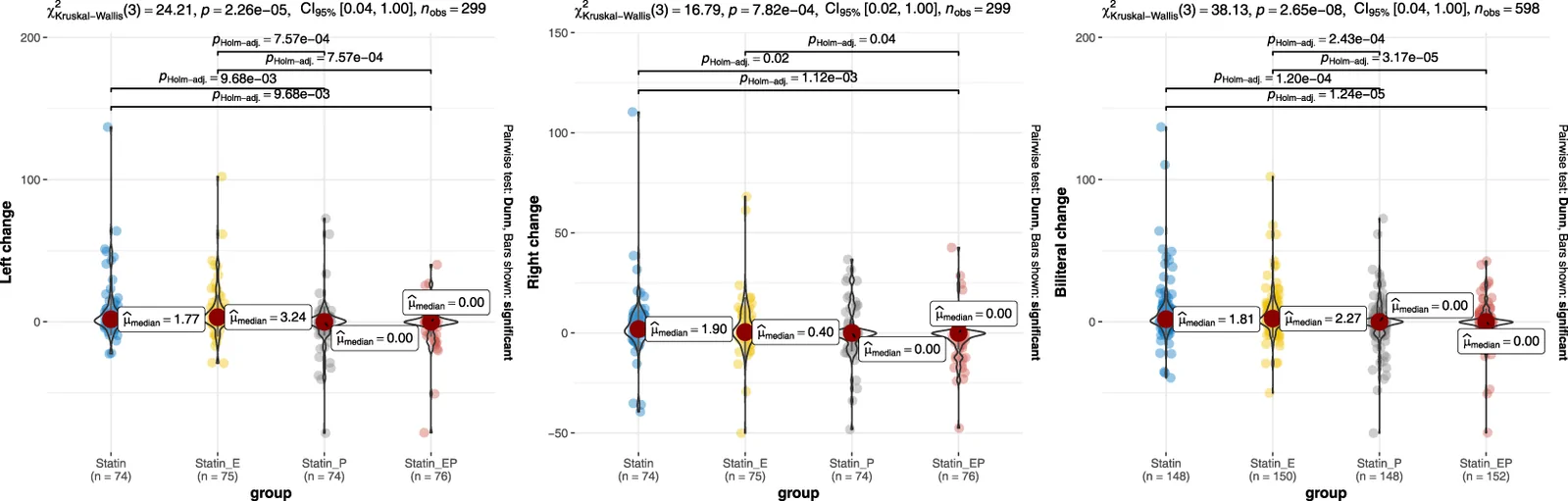
Changes in plaque sizes (post-treatment minus baseline) in the four groups.

Specifically, the changes in left carotid plaque sizes in the Statin_P and Statin_EP groups were significantly lower than that in the other groups (p = 2.39e−05, CI 95% [0.04, 1.00], Figure 6). On the right side, the changes from baseline in plaque sizes in the Statin_EP group was significantly lower than those in the Statin and Statin_E groups, and similar significant difference was found between the Statin_P and Statin groups (p = 7.82e−04, CI 95% [0.03, 1.00], Figure 6). No significant differences were found between the Statin_P and Statin_EP groups on either side (Figure 6). Similarly, the changes of bilateral plaque sizes in the Statin_P and Statin_EP groups were significantly lower than those in the other groups (p = 2.65e−08, CI 95% [0.04, 1.00], Figure 6).

**FIGURE 7:**
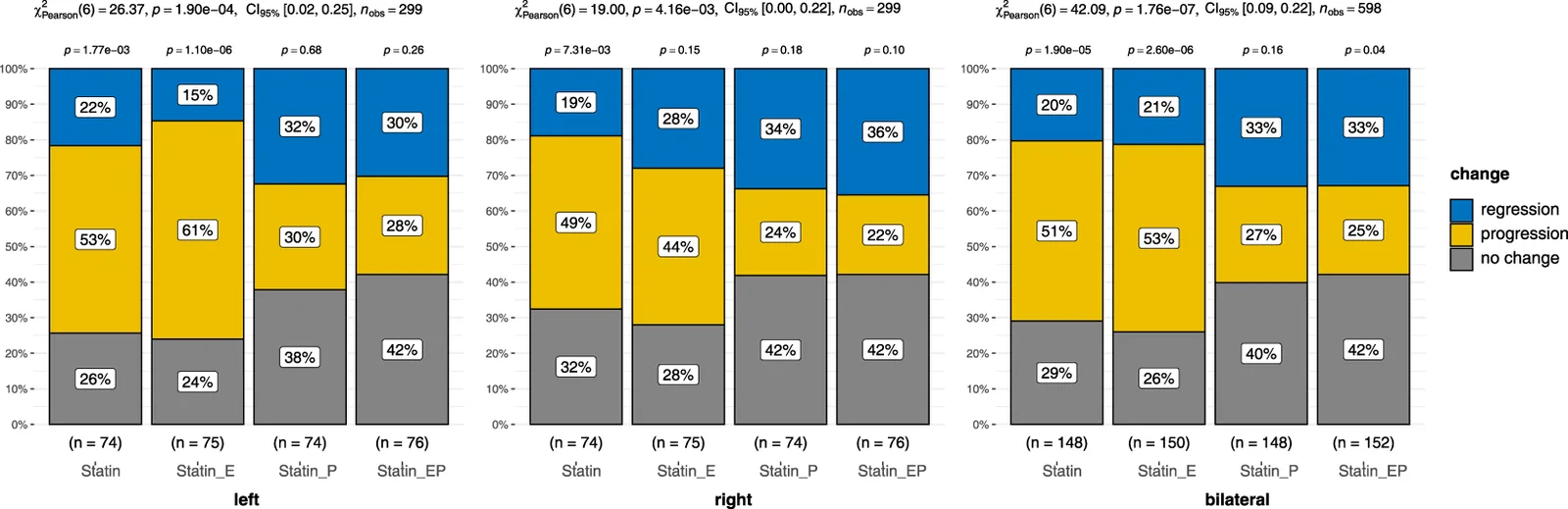
Plaque regression proportions in the four groups after treatment.

The proportions of plaque regression or progression were significantly different on either side (left: p = 1.90e−04, CI 95% [0.06, 1.00]; right: p = 4.16e−03, CI 95% [0.00, 1.00]) among the four groups after treatment. The bilateral plaque changes showed that the regression proportions in the Statin_P and Statin_EP groups exceeded 30%, while the progression proportions were lower than 30% (p = 1.76e−07, CI 95% [0.09, 0.22], Figure 7). Combination of lipid-lowering therapies with a PCSK9 inhibitor showed a greater effect on plaque regression.

The results of the mixed-effects models where the patient identifier was set as a random effect (clustering variable) to account for the correlation between plaques within the same individual were consistent with our original findings. Specifically, the Statin_EP and Statin_P arms showed significant reductions in plaque size (Wald χ^2^ = 32.79, p < 0.0001) and regression (LR χ^2^ = 28.054, p < 0.0001) compared to the Statin monotherapy group, whereas Statin_E did not (Supplementary File S1).

### 3.6 Representative patient

**FIGURE 8:**
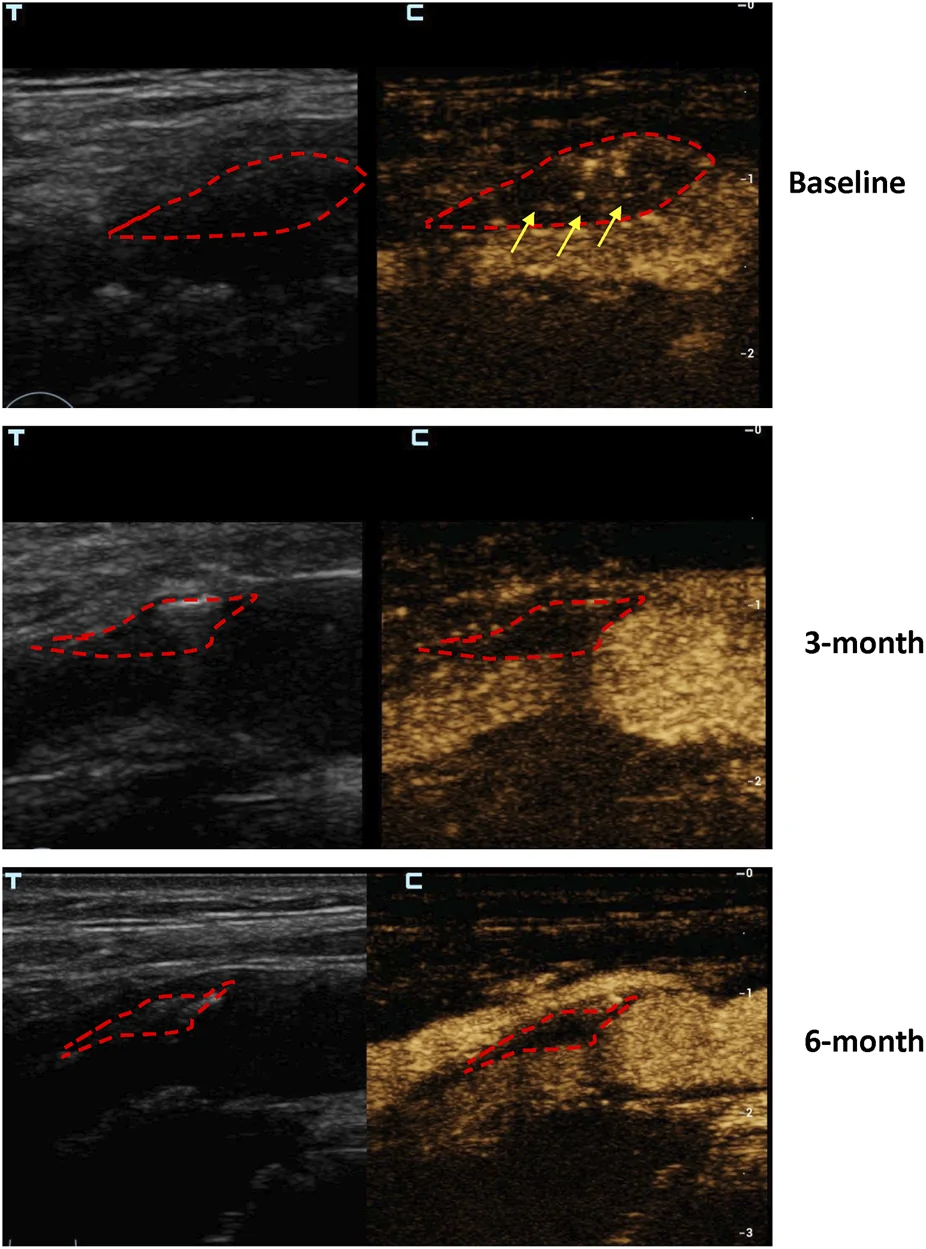
Carotid atherosclerotic plaque in a 70-year-old man at baseline, 3 months, and 6 months.

Right carotid atherosclerotic plaque in a 70-year-old man was recorded using B-mode ultrasound and CEUS (Figure 8). At baseline, the plaque at the 40th second was predominantly hypoechoic on the B-mode image, and neovessels within the plaques were evident (yellow arrows) and classified as grade 3 on CEUS imaging. After 3 months, the plaque size had decreased, and the plaque was predominantly echogenic. The microbubbles appeared confined only to the adjacent adventitial layer, classified as IPN grade 1. After 6 months, no moving microbubbles were observed in the echogenic plaque. The videos are available at https://doi.org/10.6084/m9.figshare.25997875.v3.

## 4 Discussion

### 4.1 Principal findings

This randomized open-label trial investigated the effects of different “Chinese dose” lipid-lowering therapies on the stabilization and regression of carotid plaques. After treatment, the Statin_P and Statin_EP groups showed significantly lower levels of TC and LDL-C compared to the Statin and Statin_E groups. Moderate-dose statin lipid-lowering therapies involving PCSK9 inhibitors were more effective in regressing carotid plaques and stabilizing their morphology compared to other treatments. The benefits included: 1) converting more vulnerable type I or II plaques into more stable type III or IV plaques; 2) effectively inhibiting IPN to stabilize the plaques; and 3) restraining plaque progression and even reducing plaque size. The findings underscore the efficacy of combining statin therapy with PCSK9 inhibitors, such as alirocumab, in promoting plaque stabilization and regression.

### 4.2 Comparison with previous literature

The efficacy of statins, ezetimibe, and PCSK9 inhibitors (monoclonal antibodies) in reducing serum LDL-C levels and subsequent cardiovascular events is well-documented. A European study revealed that moderate-dose statin monotherapy was the most common lipid-lowering therapy, with only 18% of very high-risk patients achieving their LDL-C goals (Ray et al., 2020). Human monoclonal antibodies, such as evolocumab and alirocumab, promote the removal of PCSK9 from circulation. This intervention decreases LDL-C levels by 60% even in individuals already on statins (Sabatine et al., 2017; Schwartz et al., 2018). The China Intensive Lipid Lowering with Statins in Acute Coronary Syndrome Study (CHILLAS) indicates that escalating the statin dosage by 1–2-fold failed to elicit a significant decrement in cardiovascular events (Zhao et al., 2014). In combination with the observation that the tolerance to high-dose statins among the Chinese population is inferior compared to that in European and American cohorts, the employment of high-intensity, high-dose statin therapy is deemed inadvisable, so the Chinese guideline for lipid management suggests that moderate-intensity statins combined with non-statin drugs can replace high-intensity statins (Li et al., 2023). Our study also supports this recommendation that combination with non-statin agents can safely reduce lipid levels of high-risk patients with better efficacy.

### 4.3 Mechanistic considerations: PCSK9 inhibition and plaque composition

Despite improvements in implementation and adherence strategies, the quest for new, effective therapies to lower LDL-C continues. The focus of lipid-lowering therapy is shifting from merely reducing lipid levels to promoting atherosclerotic plaque regression. The significant reduction in LDL-C levels observed in the Statin_P and Statin_EP groups of this study is in line with previous research highlighting the role of PCSK9 inhibitors in achieving very low LDL-C levels, which are associated with greater plaque regression (Nicho et al., 2016). This study further supports the therapeutic impact of intensive lipid-lowering therapies by showing significant decreases in carotid plaque sizes in the Statin_P and Statin_EP groups. These findings are in agreement with guidelines indicating that PCSK9 inhibitor combinations significantly reduce the progression of CVD risks in patients on statins (Mach et al., 2019). The significant plaque regression observed in the Statin_P and Statin_EP groups after 12 months of treatment is particularly encouraging, as plaque stabilization and regression is a key goal in the management of atherosclerotic disease to reduce cardiovascular events.

Beyond the regression of plaque burden, PCSK9 inhibition has also been shown to promote plaque stabilisation at the coronary level. Serial coronary imaging studies have demonstrated that PCSK9 inhibitors increase fibrous cap thickness, reduce the lipid-rich necrotic core volume and decrease plaque macrophage accumulation, shifting plaque composition toward a more stable phenotype even when absolute plaque volume reduction is modest (Biccirè et al., 2023). These compositional changes are biologically plausible given the very low on-treatment LDL-C levels achieved and provide a mechanistic complement to the LDL-C-driven regression observed in the carotid bed in the present trial. Future head-to-head carotid imaging studies with paired CEUS and MR or CT plaque characterisation will be required to confirm whether the same compositional signature occurs in carotid atherosclerosis.

These imaging techniques are driving a paradigm shift that allows for risk stratifications based on specific imaging features, such as intraplaque hemorrhage, plaque ulceration, plaque neovascularity, fibrous cap thickness, and the presence of a lipid-rich necrotic core (Choi et al., 2020). The GLAGOV trial (Nicholls et al., 2016), which investigated the use of the PCSK9 inhibitor evolocumab in combination with statins, found that patients with coronary artery disease experienced plaque regression and reduced plaque burden compared to those on statins alone. Our study demonstrated that the addition of a PCSK9 inhibitor to moderate-dose statin therapy led to greater reductions in low-density lipoprotein cholesterol (LDL-C) levels and a more pronounced stabilization of carotid plaques, as evidenced by reductions in IPN. In these studies, the use of PCSK9 inhibitors was shown to augment the lipid-lowering effects of statins, resulting in improved plaque characteristics. The importance of imaging in tracking the effects of lipid-lowering therapies on atherosclerotic plaques cannot be understated, as it enables clinicians to assess both the structural and functional changes within the plaques over time. While previous research focused primarily on coronary arteries, our study emphasizes carotid plaques, crucial for preventing cerebrovascular events. Our study advances the field of lipid-lowering therapies through a strict randomized controlled trial (RCT), directly comparing different regimens using CEUS. Unlike earlier single-arm studies, such as those by Chen et al., 2023; Lepor et al. (2021), which observed the effects of lipid-lowering therapies on carotid plaques without control groups, our trial offers stronger, comparative evidence of treatment efficacy. Furthermore, ongoing studies like the CARUSO trial (Aranzulla et al., 2021), which has yet to report results, leave a gap that our study addresses by providing timely data on PCSK9 inhibitors combined with statins for carotid plaque stabilization and regression. Advancements in vascular imaging methodologies, including computed tomography angiography (CTA), magnetic resonance angiography (MRA), and ultrasonographic imaging, have facilitated the stratification of patient risk factors.

### 4.4 Clinical implications and imaging considerations

In contrast to some coronary studies that rely on IVUS or optical coherence tomography (OCT) for assessing plaque burden and characteristics, CEUS offers a non-invasive and effective means of evaluating carotid plaque morphology, especially IPN through directly visualizing contrast agent microbubbles within plaques (Rafailidis et al., 2020). Despite the absence of IPN used for risk stratification in guidelines, emerging evidence suggests that IPN may play a critical role in identifying vulnerable plaques contributing to adverse cardiovascular outcomes (Rafailidis et al., 2020). The inner lining of the artery, known as the endothelium, becomes damaged due to factors such as high blood pressure, high cholesterol levels, smoking, aging and diabetes. This damage triggers the release of chemicals that promote the growth of new blood vessels, as the body needs to supply oxygen and nutrients to the affected area and remove waste products (Tarbell et al., 2020). However, in the context of atherosclerosis, this process can be detrimental because the newly formed blood vessels are often fragile and prone to rupture, leading to further damage to the artery, potentially causing a thrombus or clot, increasing the risk of stroke or heart attack (Goncalves et al., 2021). The mechanisms of neovascularization within atherosclerotic plaques are complex and involve multiple biological processes, including inflammation, cell death, degradation of the extracellular matrix, and the activation of endothelial and connective tissue repair responses (Lyu et al., 2021). In multiple histological and imaging studies, overall plaque burden and markers of inflammation appear to be greater in men than women and are predictive of cardiovascular events (Man et al., 2020). It is widely believed that estrogen is responsible for the protection of women from CVD in the premenopausal age group (Mathur et al., 2015). The average age of participants in our study is greater than 70 years, and this demographic difference suggests that hormonal influences, such as those seen in younger women, may not be as relevant to the gender differences in plaque stabilization observed in our cohort.

### 4.5 Strengths and limitations

This study has several limitations that should be acknowledged. Firstly, the open-label nature of the trial may introduce bias, as neither the participants nor the researchers were blinded to the treatment allocation. This could potentially influence the reporting and interpretation of results. Secondly, the study population was limited to patients from a single hospital, which may limit the generalizability of the results to broader populations with different ethnicities, comorbidities, and healthcare settings. Thirdly, the follow-up period was 6 months for plaque stabilization and 12 months for regression, which may be insufficient to capture the long-term effects of the lipid-lowering therapies on plaque stabilization and regression. Longer-term studies are needed to assess the persistent effects and potential late adverse effects. Fourthly, while CEUS provides valuable insights into plaque characteristics such as neovascularization, it remains a subjective method dependent on operator interpretation. Although we employed blinding and consensus methods to mitigate bias, we acknowledge the inherent variability in manual image interpretation. Automated and semi-automated analysis tools are emerging as promising methods for reducing subjectivity in vascular imaging, but they are not yet fully validated for routine clinical use in trials of this nature. Future research should explore the implementation of automated image analysis systems to improve objectivity. Finally, The study may not fully capture the diversity and complexity of atherosclerotic plaques, as the assessment was primarily based on ultrasound imaging, which may not detect all characteristics of plaques, such as calcification or lipid-rich necrotic cores. Therefore, the study may not fully reflect the diversity and complexity of atherosclerotic plaques. A further methodological limitation is that, although randomisation was performed at the patient level, several plaque-level analyses treated plaques within a patient as independent; a prespecified mixed-effects sensitivity analysis has been added to address within-patient clustering, but the patient-level statistical power remains limited by the original plaque-based sample-size calculation, and the corresponding confidence intervals should be interpreted cautiously.

## 5 Conclusion

This study demonstrates that combination lipid-lowering therapies, especially “Chinese dose” statin paired with PCSK9 inhibitors, are significantly more effective in both stabilizing and regressing atherosclerotic carotid plaques compared to statin monotherapy or statin plus ezetimibe. Patients receiving statin-PCSK9 combinations showed notable improvements in lipid profiles, reductions in carotid plaque size, and conversion of vulnerable plaques to more stable forms. These findings highlight the potential of statin-based combination therapies for reducing CVD risk in high-risk populations, particularly among Asian patients where lower statin doses may be preferable.

## Data Availability

The datasets presented in this study can be found in online repositories. The names of the repository/repositories and accession number(s) can be found below: https://doi.org/10.6084/m9.figshare.25997875.v3.
