# Supplementary material for: Effects of different statin-based lipid-lowering therapies on stabilization and regression of carotid plaque: a randomized open-label trial
Source: Front Pharmacol. 2026 Aug 27;17:1841158. doi: 10.3389/fphar.2026.1841158 (PMC13558017; doi:10.3389/fphar.2026.1841158)
Supplement: Supplementary file 1 [file DataSheet1.pdf]

Carotid Plaque Treatment — Mixed-effects Ordinal Logistic Regression

Response: `dchange` (ordered: regression → no change → progression)
Fixed factor: `group`
Random factor: `patient` (random intercept; 2 plaques per patient)

---

0. Method Rationale

`dchange` is an **ordered 3-level categorical outcome** and the observations are **clustered within patients**. Two simple but inappropriate alternatives:

- A chi-square / Fisher test on the cross-tabulation **ignores within-patient correlation** and would treat 598 plaque observations as independent.
- A standard proportional-odds regression (e.g. `polr`) **ignores the patient random effect**.

The appropriate model is therefore a **cumulative logit mixed model** (`ordinal::clmm`), which:

1. respects the natural ordering of the outcome,
2. accounts for the within-patient correlation via a random intercept,
3. uses adaptive Gauss–Hermite quadrature to integrate out the random effects.

A Brant test is reported to check the proportional-odds assumption; a nominal (multinomial) model is fitted as a sensitivity analysis if the assumption is violated.

---

1. Sample Size and Outcome Distribution

Group	Plaques (n)	regression	no change	progression
Statin	148	30 (20.3%)	43 (29.1%)	75 (50.7%)
Statin_E	150	32 (21.3%)	39 (26.0%)	79 (52.7%)
Statin_EP	152	50 (32.9%)	64 (42.1%)	38 (25.0%)

Statin_P	148	49 (33.1%)	59 (39.9%)	40 (27.0%)
**Total**	**598**	**161 (26.9%)**	**205 (34.3%)**	**232 (38.8%)**

Note the inverted pattern in the Statin_EP and Statin_P arms: a markedly lower proportion of progressions and a higher proportion of regressions/no-change.

---

2. Cumulative Logit Mixed Model

`clmm(dchange ~ group + (1 | patient), link = "logit", nAGQ = 7)`

2.1 Random effects

Group	Variance	SD
patient (Intercept)	1.041	1.020

The patient-level variance is non-trivial, confirming the need to account for clustering.

2.2 Overall effect of `group` (Likelihood-ratio test)

Model	df	AIC	logLik	LR χ^2	df	p
Null (intercept + patient RE)	3	1286.9	−640.47	—	—	—
Full (with `group`)	6	1264.9	−626.44	28.054	3	**3.54×10^{-6}**

The four arms differ significantly in their outcome distribution.

2.3 Fixed-effect coefficients and odds ratios (reference: Statin)

In a cumulative logit model, the **OR < 1 means lower odds of being in a worse category** (progression) versus being in a better one (regression / no change). Equivalently, OR < 1 → the group has a more favourable outcome distribution.

Term	Estimate	SE	z	p	OR	OR 95% CI
groupStatin_E	0.041	0.292	0.140	0.888	1.042	(0.588, 1.847)
groupStatin_EP	−1.080	0.290	−3.726	**1.94×10^{-4}**	0.340	(0.193, 0.599)

groupStatin_P	−1.045	0.292	−3.574	**3.51×10^{-4}**	0.352	(0.198, 0.624)

Thresholds

Threshold	Estimate	SE	z	p
regression → no change	−1.795	0.238	−7.553	$< 10^{-13}$
no change → progression	0.035	0.210	0.165	0.869

The first threshold is well identified (clear separation between regression and the rest). The second is close to zero, meaning on the logit scale there is roughly a 50/50 split between "no change or below" and "progression" in the reference group.

2.4 Predicted outcome probabilities (population-averaged)

Group	P(regression)	P(no change)	P(progression)
Statin	0.142	0.366	0.491
Statin_E	0.137	0.361	0.502
Statin_EP	0.328	0.425	0.247
Statin_P	0.321	0.426	0.254

The two PCSK9-inhibitor arms cut the probability of progression roughly **in half** (~25% vs ~50%) and double the probability of regression.

---

3. Pairwise Comparisons (Holm-adjusted)

Each sub-model is a `clmm(dchange ~ group + (1 | patient))` fit to two arms at a time; p-values are adjusted with the Holm method.

Comparison	Estimate	SE	z	p (raw)	OR	OR 95% CI	p (Holm)	Sig.
Statin – Statin_E	0.039	0.272	0.142	0.887	1.040	(0.609, 1.773)	1.000	ns

Statin – Statin_EP	−1.146	0.316	−3.623	2.91×10^{-4}	0.318	(0.171, 0.591)	**0.0014**	**
Statin – Statin_P	−0.949	0.258	−3.683	2.31×10^{-4}	0.387	(0.234, 0.641)	**0.0014**	**
Statin_E – Statin_EP	−1.238	0.344	−3.595	3.24×10^{-4}	0.290	(0.148, 0.569)	**0.0014**	**
Statin_E – Statin_P	−1.026	0.281	−3.656	2.56×10^{-4}	0.359	(0.207, 0.621)	**0.0014**	**
Statin_EP – Statin_P	0.038	0.302	0.127	0.899	1.039	(0.574, 1.880)	1.000	ns

Significant contrasts

- Statin vs Statin_EP, Statin vs Statin_P, Statin_E vs Statin_EP, Statin_E vs Statin_P — all Holm-adjusted $p = 0.0014$

Non-significant

- Statin ≈ Statin_E ($p = 1.000$): adding E alone does not change the outcome distribution
- Statin_EP ≈ Statin_P ($p = 1.000$): the two PCSK9-inhibitor arms are statistically indistinguishable

---

4. Proportional-Odds Assumption (Brant Test)

`brant::brant()` applied to a `polr` fit on the same data (the random effect is omitted in the Brant test, so the test is approximate).

**Term**	**χ^2**	**df**	**p**
Statin_E	0.32	1	0.572
Statin_EP	3.01	1	0.083
Statin_P	1.75	1	0.186
**Omnibus**	**7.23**	**3**	**0.065**

The proportional-odds assumption is **not strongly violated** (omnibus p = 0.065), but the Statin_EP coefficient is borderline (p = 0.083). The ordinal model is therefore acceptable, and the alternative nominal model is reported below as a sensitivity check.

---

5. Sensitivity Analysis — Nominal (Multinomial) Logistic Regression

Ignoring the ordering and clustering, a `multinom` model produces broadly the same picture.

Outcome contrast	Coef. (Statin_EP)	Coef. (Statin_P)
no change vs regression	−0.113	−0.174
progression vs regression	**−1.191**	**−1.119**

The PCSK9-inhibitor arms significantly reduce the log-odds of being in the **progression** category (vs regression) by about 1.1–1.2 units, while leaving the no-change-vs-regression log-odds essentially unchanged. This mirrors the cumulative model: the treatment effect acts primarily on the upper end of the outcome scale.

---

6. Key Takeaways

1. **Outcome distribution differs significantly across groups** (LR $\chi^2 = 28.05$, $p = 3.5 \times 10^{-6}$).
2. **Adding E alone is ineffective**: Statin ≈ Statin_E (OR = 1.04, p = 0.89).
3. **PCSK9-inhibitor-containing regimens are highly effective**:
    - Statin_EP OR = 0.34 (95% CI 0.19 – 0.60), p = 0.0002
    - Statin_P OR = 0.35 (95% CI 0.20 – 0.62), p = 0.0004
4. **EP and P are equivalent** (p = 0.90): the benefit in Statin_EP is driven by the P component.
5. **Effect is concentrated in preventing progression**: probability of progression drops from ~50% (Statin / Statin_E) to ~25% (Statin_EP / Statin_P), while the no-change probability rises modestly.
6. **Patient-level heterogeneity is real** (SD of patient intercept ≈ 1.02 on the logit scale), so the mixed model is appropriate.

Generated by `plaque1.r` • R 4.6.0 • `ordinal::clmm` / `brant` / `nnet::multinom`

Carotid Plaque Treatment — Statistical Analysis Report (Non-parametric)

0. Rationale for Method Selection

Standard parametric MLM (`lmer`) with Tukey-adjusted pairwise comparisons requires both **normality of residuals** and **homogeneity of variances**. Formal checks (see § 3) showed that residuals deviate significantly from normality, so the Tukey post-hoc test based on the t-distribution is not appropriate. Therefore:

Analysis	Method	Reason
**Primary**	Kruskal–Wallis rank test on per-patient mean `dvalue`	Distribution-free, robust to skew and heavy tails
**Pairwise**	Dunn's test (Bonferroni-adjusted)	Standard non-parametric post-hoc for KW; controls family-wise error
**Sensitivity**	Robust Mixed Model (`robustlmm::rlmer` , smoothed Huber)	Keeps mixed-model structure with down-weighted outliers

> Each patient contributes 2 plaques; therefore we aggregated `dvalue` to the patient level (`mean(dvalue)` per patient) for the primary analysis. This yields one independent observation per patient (n = 299) and avoids pseudo-replication. `dvalue` = the change of plaque size (post-treatment minus baseline)

---

1. Sample Size by Group

Group	Patients (n)	Plaques (n)
Statin	74	148
Statin_E	75	150

Statin_EP	76	152
Statin_P	74	148
**Total**	**299**	**598**

2. Descriptive Statistics

2.1 Per-patient (analysis unit for primary analysis)

Group	n (patients)	Median `dvalue`	Q1	Q3	IQR
Statin	74	2.11	0.02	6.95	6.94
Statin_E	75	2.82	−0.54	7.89	8.43
Statin_EP	76	−0.34	−3.79	2.22	6.02
Statin_P	74	0.00	−5.22	3.72	8.95

2.2 Per-plaque (reference)

Group	n	Mean	SD	Median	Min	Max
Statin	148	5.77	19.5	1.81	−39.4	137.0
Statin_E	150	4.86	16.4	2.27	−50.1	102.0
Statin_EP	152	−1.31	13.0	0.00	−78.0	42.6
Statin_P	148	−0.58	15.8	0.00	−78.3	72.7

3. Assumption Checks

Test	Statistic	df	p	Conclusion
Shapiro–Wilk (MLM residuals)	W = 0.836	—	$< 2.2 \times 10^{-16}$	**Normality violated**

Levene (median-centered) on dvalue ~ group	F = 0.912	3, 594	0.435	Variance homogeneity not rejected

The residuals from the parametric MLM clearly deviate from a normal distribution. The Levene test does not reject equal variances, but given the strong normality violation, a non-parametric approach is preferred for robustness.

---

4. Primary Analysis — Kruskal–Wallis

H_0: the four groups have the same distribution of `dvalue`.
Test: Kruskal–Wallis rank-sum test on per-patient mean `dvalue`.

Statistic	Value
χ^2 (Kruskal–Wallis)	26.528
df	3
p-value	**7.40×10^{-6}**
ε^2 (epsilon-squared effect size)	0.0798

Conclusion: The null hypothesis of identical distributions is rejected ($p < 0.001$). Epsilon-squared ≈ 0.08 indicates a small-to-medium effect (Cohen's rough guideline: 0.01 small, 0.06 medium, 0.14 large).

---

5. Pairwise Comparisons — Dunn's Test (Bonferroni-adjusted)

Effect size reported as **rank-biserial $r = Z / \sqrt{n}$** ($n = 299$ patients).

Comparison	Z	p (unadj.)	p (adj.)	r	Sig.
Statin − Statin_E	0.458	0.647	1.000	0.027	ns
Statin − Statin_EP	4.138	3.50×10^{-5}	**2.10×10^{-4}**	0.239	***
Statin − Statin_P	3.509	4.50×10^{-4}	**2.70×10^{-3}**	0.203	**

Statin_E − Statin_EP	3.691	2.24×10^{-4}	**1.34×10^{-3}**	0.213	**
Statin_E − Statin_P	3.062	2.19×10^{-3}	**1.32×10^{-2}**	0.177	*
Statin_EP − Statin_P	−0.606	0.545	1.000	−0.035	ns

Significant contrasts (Bonferroni p < 0.05)

- **Statin vs Statin_EP**: p = 0.00021, r = 0.24 (small-medium effect)
- **Statin vs Statin_P**: p = 0.0027, r = 0.20 (small effect)
- **Statin_E vs Statin_EP**: p = 0.0013, r = 0.21 (small-medium effect)
- **Statin_E vs Statin_P**: p = 0.0132, r = 0.18 (small effect)

Non-significant

- Statin ≈ Statin_E (p = 1.000): adding E alone does not shift the distribution
- Statin_EP ≈ Statin_P (p = 1.000): EP and P arms are equivalent

---

6. Sensitivity Analysis — Robust Mixed Model

To verify the non-parametric findings while preserving the repeated-measures structure, a robust MLM was fit using `robustlmm::rlmer` (smoothed Huber weighting; down-weights 145/598 ≈ 24% of observations).

Random effects: residual variance = 65.5 (SD = 8.09); patient-level variance ≈ 0 (all down-weighted to 1) → between-patient differences appear to be driven by outliers that the robust estimator correctly de-emphasises.

Fixed effects (Wald z-tests)

Term	Estimate	Robust SE	z	p	Sig.
(Intercept) Statin	3.148	0.682	4.615	3.93×10^{-6}	***
groupStatin_E	−0.043	0.961	−0.045	0.964	ns
groupStatin_EP	−4.075	0.958	−4.252	2.12×10^{-5}	***
groupStatin_P	−3.721	0.965	−3.857	1.15×10^{-4}	***

Overall group effect (Wald χ^2) = 32.79, df = 3, **p < 0.0001**.

The robust model reaches the same qualitative conclusion as the non-parametric test: Statin_EP and Statin_P significantly reduce `dvalue` vs Statin monotherapy, while Statin_E does not.

---

7. Summary of Findings

Question	Answer
Do the four arms differ?	**Yes** — Kruskal–Wallis $\chi^2 = 26.53$, $p = 7.4 \times 10^{-6}$
Does adding E help?	**No** — Statin ≈ Statin_E (p = 1.000)
Does adding P help?	**Yes** — Statin > Statin_P (p = 0.0027)
Does adding EP help?	**Yes** — Statin > Statin_EP (p = 0.00021)
Is EP better than P?	**No** — Statin_EP ≈ Statin_P (p = 1.000)
Is EP better than E alone?	**Yes** — Statin_E > Statin_EP (p = 0.0013)

Interpretation: The PCSK9-inhibitor-containing regimens (Statin_EP and Statin_P) yield a significantly smaller change in plaque size compared with Statin monotherapy or Statin + E. Statin_EP and Statin_P are statistically indistinguishable, suggesting the added benefit in the EP arm is driven by the P component rather than the E component.

Generated by `plaque.r` · R 4.6.0 · `rstatix` / `FSA` / `robustlmm`
